# Investigation of the Effects of Perfluorooctanoic Acid (PFOA) and Perfluorooctane Sulfonate (PFOS) on Apoptosis and Cell Cycle in a Zebrafish (*Danio rerio*) Liver Cell Line

**DOI:** 10.3390/ijerph121215012

**Published:** 2015-12-09

**Authors:** Yuan Cui, Wei Liu, Wenping Xie, Wenlian Yu, Cheng Wang, Huiming Chen

**Affiliations:** Chinese Academy of Inspection and Quarantine, Beijing 100123, China; cuiy@aqsiqch.ac.cn (Y.C.); liuw@aqsiqch.ac.cn (W.L.); xiewp@aqsiqch.ac.cn (W.X.); yuwl@aqsiqch.ac.cn (W.Y.); wangz@aqsiqch.ac.cn (C.W.)

**Keywords:** perfluorooctanoic acid (PFOA), perfluorooctane sulfonate (PFOS), zebrafish liver cells (ZFL), apoptosis

## Abstract

This study aimed to explore the effects of perfluorooctanoic acid (PFOA) and perfluorooctane sulfonate (PFOS) on apoptosis and cell cycle in a zebrafish (*Danio rerio*) liver cell line (ZFL). Treatment groups included a control group, PFOA-IC_50_, PFOA-IC_80_, PFOS-IC_50_ and PFOS-IC_80_ groups. IC_50_ and IC_80_ concentrations were identified by cellular modeling and MTT assays. mRNA levels of p53, Bcl-2, Bax, Caspase-3 and NF-κB p65 were detected by qPCR. Cell apoptosis and cell cycle were detected by flow cytometry and the protein levels of p53, Bcl-2, Bax, Caspase-3 and NF-κB p65 were determined by western blotting. Both PFOA and PFOS inhibited the growth of zebrafish liver cells, and the inhibition rate of PFOS was higher than that of PFOA. Bcl-2 expression levels in the four groups were significantly higher than the control group and Bcl-2 increased significantly in the PFOA-IC_80_ group. However, the expression levels of Bax in the four treatment groups were higher than the control group. The percentage of cell apoptosis increased significantly with the treatment of PFOA and PFOS (*p* < 0.05). Cell cycle and cell proliferation were blocked in both the PFOA-IC_80_ and PFOS-IC_80_ groups, indicating that PFOA-IC_80_ and PFOS-IC_50_ enhanced apoptosis in ZFL cells.

## 1. Introduction

Multicellular animals dispose of unneeded or damaged cells in an ordered fashion by apoptosis [1]. Apoptosis is a programmed cell death pathway that removes damaged cells to protect the body, and it is especially important in vertebrate embryonic development [2]. The relationship between stress-induction and apoptosis during the development has been confirmed by experimental studies using zebrafish (*Danio rerio*) embryos, and researches on apoptotic genes, including the Bcl-2 gene family, p53, and the caspase family, are still under the spotlight [3,4,5,6]. Many apoptosis related genes in mammals were first identified in zebrafish, which implies the high degree of conservation within the apoptosis pathway across species [7]. Accordingly, zebrafish is considered to be an effective animal model to reveal the relationship between stress-induction and apoptosis.

Perfluorinated chemicals (PFCs) are synthesized fluorinated compounds composed of a carbon backbone that is typically 4–14 atoms in length and a charged moiety, primarily a carboxylate or sulfonate. Perfluorooctanoic acid (PFOA) and perfluorooctane sulfonate (PFOS) are two of the most widely used PFCs. PFOA and PFOS have already been considered as emerging persistent organic pollutants [8]. The distribution of PFOA and PFOS in the environment has increased universally in recent years [9] and their accumulation and toxic effects have become serious issues to be addressed [10]. PFOS and PFOA are important PFCs with hydrophobic and oleophobic properties [11], which are widely used in carpet, leather, paper, and fiber manufacture, as well as the textile industry and other consumer products.

Previous studies have reported on the toxicology of PFOA and PFOS. PFOA and PFOS can be found in the liver, serum and other tissues of humans and animals [12]. For rats chronic exposure to PFOA and PFOS is associated with tumor development in the liver, pancreas and testis [13,14,15]. Furthermore, PFOS has been reported to induce *in vivo* hepatocellular hypertrophy, lipid vacuolation, loss of body weight, and lower serum total cholesterol in monkeys [16]. Guruge *et al.* found that gene regulation related with cell communication, growth and apoptosis, and fatty acid metabolisms in rats after exposure to PFOA [17]. In addition, PFOA had a toxic effect on the liver, not on the kidney, in mice exposed to PFOA in their drinking water [18].

Mechanistically, some reports showed that PFOA induced apoptosis in a dose- and time-dependent manner in hepatoma HepG2 cells [19,20]. Moreover, genotoxic risk and oxidative DNA damage was reported in HepG2 cells exposed to PFOA [21]. Similarly, Liu *et al.* demonstrated that PFOA and PFOS were individually able to produce oxidative stress and induce apoptosis through the involvement of caspases in primary cultured tilapia (*Oreochromis niloticus*) hepatocytes [22].

However, there has been no investigation into the effects of PFOA and PFOS on apoptosis in the zebrafish liver cell line (ZFL). In this study, accordingly, we aimed to detect the mRNA and protein levels of apoptosis related genes in ZFL and to explore the mechanisms how PFOA and PFOS mediate apoptosis in ZFL.

## 2. Materials and Methods

### 2.1. ZFL Culture

ZFL was obtained from American Type Culture Collection (ATCC, Rockville, MD, USA). The cell line was cultured in a medium consisted of 50% Leibovitz L-15 (Gibco BRL Co. Ltd. Gaithersburg, MD, USA), 15% Ham F-12 (Gibco) and 35% Dulbecco’s modified eagle medium (DMEM; Gibco), supplemented with 50 ng/mL epidermal growth factor (EGF; Gibco), 0.01 mg/mL insulin (Sigma), 15 mM 4-(2-hydroxyethyl)-1-piperazineethanesulfonic acid (HEPES; Gibco) and 5% fetal bovine serum (FBS; Gibco) at 37 °C and saturated with 5% CO_2_ in a humidified atmosphere. Next, 10% dimethyl sulfoxide (DMSO) and 90% FBS were suspended as cell freezing medium. All the ZFL cells were recovered in a conventional method and observed [23]. The ZFL cells were seeded on 96-well plates with a cell density of 1 × 10^4^ per well and incubated overnight to achieve a 85% confluence, and then the cells were sub-cultured and frozen.

### 2.2. Cellular Modeling and MTT Assay

50% inhibitory concentration (IC_50_) and 80% inhibitory concentration (IC_80_) were measured by MTT assays. ZFL cells were treated with 100, 50, 25, 12.5, 6.25, 3.125, 1.062, 0.53, 0.2605, 0.130, 0.065 and 0 μg/mL of PFOA or PFOS for 48 h, respectively. Cells were incubated for 4 h after 100 μL serum-free medium and 10 μL MTT were added. ODs were detected at 490 nm after the 100-μL solubilization buffer (10% SDS + 0.01M HCl) was added.

### 2.3. Quantitative Real Time-Polymerase Chain Reaction (qPCR)

Total RNA was extracted using a RNA Extraction Kit according to the manufacturer’s instructions (CWbio, Beijing, China). Reverse transcription was performed by HiFi-MMLVcDNA (KITCWbio. Co. Ltd., Beijing, China). The PCR conditions were as follows: 45 cycles at 95 °C for 10 min, 95 °C for 15 s and 60 °C for 60 s successively. β-Actin was used as an internal control in the experiment. All reactions were completed in triplicate using independently-extracted RNA and the relative expression levels were detected by fluorescence ratio PCR instrument (ABI 7500) and calculated using the 2^−ΔΔ*C*t^ comparative CT method. Gene-specific primers for qPCR are listed in Table 1.

### 2.4. Flow Cytometry of Cell Apoptosis Detection

Annexin V-FITC-PI kit (Beijing 4A Biotech Co., Ltd., Beijing, China) was used for detecting cell apoptosis by flow cytometry. Annexin V is a Ca^2+^-dependent phospholipid-binding protein that has a high affinity for phospholipid phosphatidylserine (PS), and is useful for identifying apoptotic cells with exposed PS. Propidium iodide (PI) is a standard flow cytometric viability probe and is used to distinguish viable from nonviable cells, as intact membranes of viable cells exclude PI while membranes of dead or damaged cells are permeable to PI. Briefly, single-cell suspension of 1 × 10^6^ cells was added into a 1.5-mL centrifuge tube, centrifuged at 300× *g* for 5 min, and the supernatant was discarded. Afterwards, 1 mL of PBS was added to the cell pellet, and the samples were centrifuged at 300× *g*. This above washing procedure was repeated twice. Subsequently, 100 μL of binding buffer, 5 μL Annexin V-FITC and 10 μL PI were added and the samples were incubated at room temperature for 15 min, ensuring no exposure to light. Finally, 400 μL binding buffer was added to the samples and cell apoptosis was detected by flow cytometry. A software package called Cellquest was used to analyze cell apoptosis.

**Table 1:** Primer sequences for RT-PCR.

Primer	Sequences (5′ to 3′)	PCR Fragments (bp)
P53 forward primer	CCACCATGAGAGAACACCTGAT	144
P53 reverse primer	GCTGAGGAGCTTCATAGAGAACC	
Bcl-2 forward primer	TGGGCTCATCTCCTTCTCC	178
Bcl-2 reverse primer	TCTCCTCACAGCTCCACATC	
Bax forward primer	GGAGGCGATACGGGCAGT	151
Bax reverse primer	TTGCGAATCACCAATGCTGTG	
Caspase-3 forward primer	ACTGATGGTTCTGTGGAGC	200
Caspase-3 reverse primer	CGCATAGAGGAAGTCTGCTT	
NFKBNF-κB p65 forward primer	AAGATGAGAACGGAGACACGC	404
NFKBNF-κB p65 reverse primer	TACCAGCAATCGCAAACAACG	
B-actin forward primer	CAGGGCGTGATGGTGGGGAT	226
β-actin reverse primer	GGTTGGCTTTGGGGTTGAG	
GAPDH forward primer	AGTTGTAAGCAATGCCTCCTG	191
GAPDH reverse primer	CTGGGATGATGTTCTGACTGG	
B2M forward primer	GCACTCATCACTTTTGCACTTC	135
B2M reverse primer	GCTCACATAGCAGATCAGGGT	

### 2.5. Cell Cycle Analysis

4 × 10^5^ cells were seeded into 6-well plates, synchronized by serum starvation for 24 h, and re-entered into the cell cycle by an exchange of a medium with 10% FBS DMEM for 24 h. Both adherent and non-adherent cells were harvested and fixed in 70% ethanol at 4 °C overnight. Cells were incubated with RNase A at 37 °C for 30 min and then stained with PI. Cell cycle status was measured by flow cytometry.

### 2.6. Western Blot Assay

Cells were lysed with RIPA buffer and the proteins lysates were separated using 12% SDS-PAGE. The proteins were transferred onto a PVDF membrane for 1 h and then the membrane was incubated with primary antibody overnight at 4 °C after being blocked with 3% BSA-TBST solution for 30 min. The membrane was washed with TBST solution three times, 10 min each time. Then the membrane was incubated with secondary antibody (Beijing TDY Biotech Co., Ltd., Beijing, China) for 2 h at room temperature. Finally, the membrane was developed after being washed again with TBST solution three times, 10 min each time. The protein expressions of p53 (Beijing TDY Biotech Co., Ltd.), Caspase 3 (Abcam, Cambridge, UK) and Bcl-2 (Beijing TDY Biotech Co., Ltd.) were detected by western blotting. GAPDH (Beijing TDY Biotech Co., Ltd.) and B2M (Beijing TDY Biotech Co., Ltd.) were used as an internal control for the experiment.

### 2.7. Statistical Analysis

Statistical analysis was performed using the software package SPSS 13.0 (SPSS, Inc., Chicago, IL, USA) Data are represented as the mean value and statistical significance was determined by one-way ANOVA. Differences were considered significant at *p* < 0.05.

## 3. Results

### 3.1. Detection of IC_50_ and IC_80_ Concentrations

**Figure 1:**
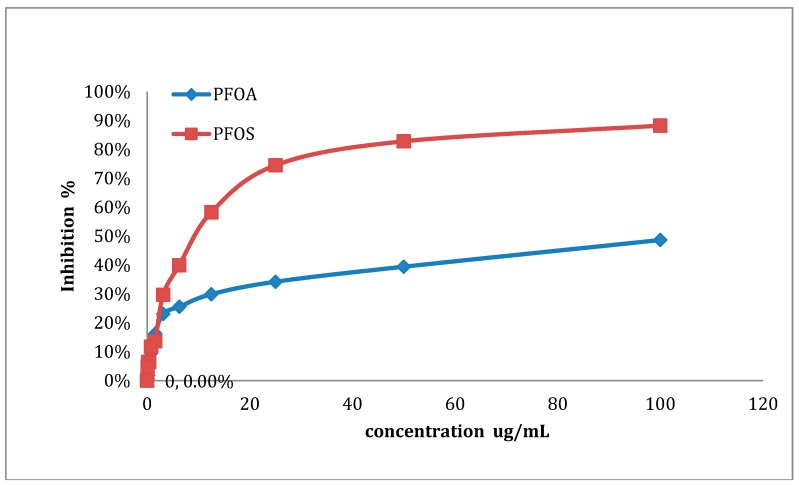
Inhibition rate of PFOS and PFOA in ZFL cells. ZFL cells were treated with 100 μg/mL, 50, 25, 12.5, 6.25, 3.125, 1.062, 0.53, 0.2605, 0.130, 0.065 or 0 μg/mL of either PFOA or PFOS for 48 h. The inhibition rate was determined by MTT assay.

To determine the IC_50_ and IC_80_ concentrations of PFOA and PFOS, ZFL cells were treated with different concentrations of PFOA or PFOS, and the inhibition rate was determined by an MTT assay (see Figure 1). As shown in Figure 1, both PFOA and PFOS could inhibit zebrafish liver cells. Moreover, the inhibition rate of PFOS was higher than that of PFOA. As summarized in Table 2, the IC_50_ and IC_80_ concentrations of PFOA were 84.76 μg/mL and 150.97 μg/mL, respectively (the IC_80_ for PFOA was extrapolated) The IC_50_ and IC_80_ concentrations of PFOS were 27.92 μg/mL and 56.77 μg/mL, respectively. The subsequent experiments were performed on IC_50_ and IC_80_ samples of PFOA and PFOS.

**Table 2:** IC_50_ and IC_80_ of PFOA and PFOS.

Substances	IC_50_	IC_80_
PFOA (μg/mL)	84.76	150.97
PFOS (μg/mL)	27.92	56.77

### 3.2. Relative Expression of p53, Bcl-2, Bax, Caspase-3 and NFκB p65

**Figure 2:**
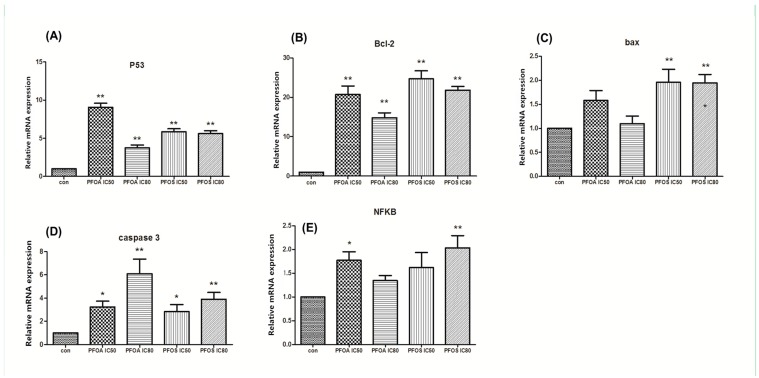
Relative gene expression of p53, Bcl-2, Bax, Caspase-3 and NF-κB p65. ZFL cells were treated with PFOA or PFOS at IC_50_ or IC_80_ concentrations for 48 h. β-actin was used as internal control. The experiment was repeated three times. * *p* < 0.05, ** *p* < 0.01.

qPCR was performed to determine whether PFOA and PFOS treatment affected the expression of apoptosis-related genes, such as p53, Bcl-2, Bax, Caspase-3 and NF-κB p65,. As shown in Figure 2, different relative levels of p53, Bcl-2, Bax, Caspase-3 and NF-κB p65 mRNA levels were induced upon treatment with PFOA-IC_50_, PFOA-IC_80_, PFOS-IC_50_ and PFOS-IC_80_ in ZFL cells. Bcl2 expression increased compared to the control group after PFOA and PFOS treatment, with the PFOS-IC_50_ group exhibiting the highest induction. p53 had the highest expression in the PFOA-IC_50_ group with an 8.709-fold induction. The mRNA level of Bax was induced with the treatment of PFOS, but was not induced with treatment of PFOA, and the PFOS-IC_50_ and PFOS-IC80 groups exhibited comparable induction of Bax. For Caspase-3, the highest expression level was induced in the PFOA-IC_80_ group. NF-κB p65 expression level was induced in all groups except the PFOA-IC_80_ group. These results showed that PFOA and PFOS treatment could affect the expression of apoptosis related genes, including p53, Bcl-2, Bax, Caspase-3 and NF-κB p65.

### 3.3. Cell Apoptosis and Cycle in ZFL Cells

**Figure 3:**
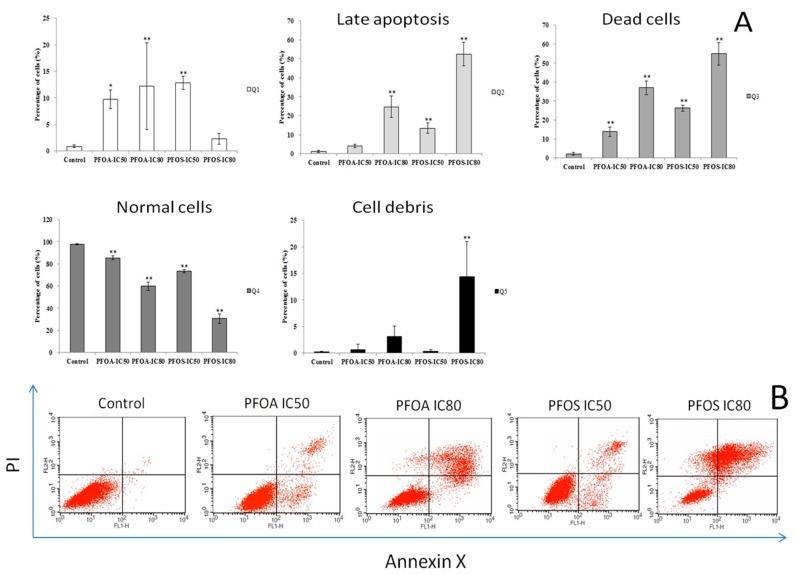
Detection of apoptosis on ZFL cells exposed to PFOA and PFOS using flow cytometry. ZFL cells were treated with PFOA or PFOS at IC_50_ or IC_80_ concentrations for 48 h. Annexin V-FITC-PI kit was used for detecting cell apoptosis by flow cytometry. Percentage of cell apoptosis after treatment with PFOA or PFOS in three independent experiments. * *p* < 0.05, ** *p* < 0.01.

Flow cytometry was used to detect the effects of PFOA and PFOS on apoptosis in ZFL cells. As shown in Figure 3, the percentage of cell apoptosis increased significantly after treatment with PFOA or PFOS in three independent experiments (*p* < 0.05). In addition, Figure 3 shows that the early apoptosis rate increased significantly in the PFOA-IC_50_, PFOA-IC_80_ and PFOS-IC_50_ groups. Moreover, significant promotion of late apoptosis was also observed in the PFOA-IC_80_, PFOS-IC_50_ and PFOS-IC_80_ groups.

**Figure 4:**
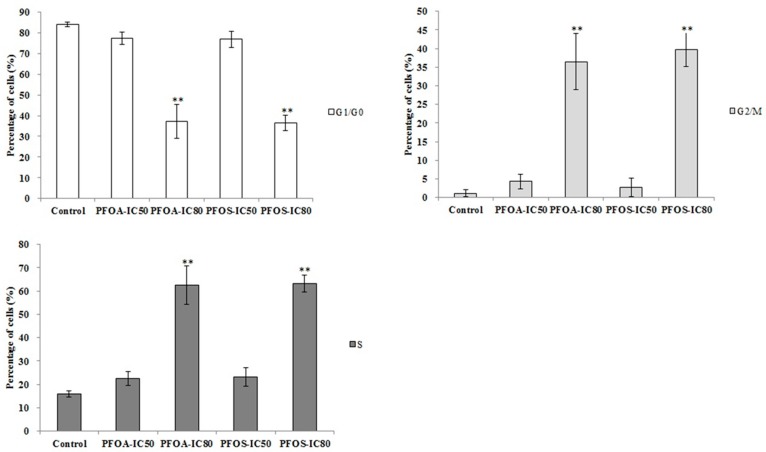
Cell cycle of ZFL under the inducing of PFOA and PFOS. The experiment was repeated three times. ** *p* < 0.01.

As shown in Figure 4, compared with the control group, the percentage of cells in G1/G0 phase decreased significantly (*p* < 0.01), and the percentage of cells in G2/M phase and S phase increased significantly (*p* < 0.01) in both the PFOA-IC_80_ group and the PFOS-IC_80_ group. The results indicated that cell proliferation was blocked in both the groups.

### 3.4. Protein Expression Levels of p53, Bcl-2, and Active-Caspase 3 in ZFL Cells Induced with PFOA and PFOS

**Figure 5:**
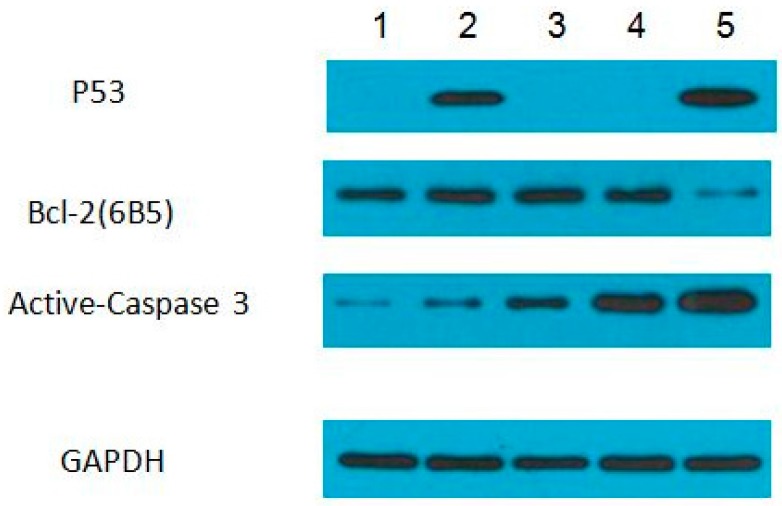
Protein expression level of P53, Bcl-2 and Caspase-3. 1, Control group; 2, PFOA-IC_50_ group; 3, PFOA-IC_80_ group; 4, PFOS-IC_50_ group; 5, PFOS-IC_80_ group. ZFL cells were treated with PFOA or PFOS at IC_50_ or IC_80_ concentrations for 48 h. The protein expressions of p53, caspase 3 and Bcl-2 were detected. GAPDH was used as an internal control.

To determine the protein levels of p53, Bcl-2, and active-caspase 3 in PFOA or PFOS treated ZFL cells, western blotting was performed. As shown in Figure 5, p53 protein was expressed in PFOA-IC_50_ and PFOS-IC_80_ groups and Bcl2 was expressed in all groups, in spite of a lower expression in the PFOS-IC_80_ group. For active-caspase 3, the expression in PFOA-IC_50_, PFOA-IC_80_, PFOS-IC_50_ and PFOS-IC_80_ groups were comparable.

## 4. Discussion

Although zebrafish is a mostly-widely model organism in laboratories, zebrafish cell lines are still unexploited and limited in applications, partly due to their unknown genetic and physiological properties. In this study, ZFL cells were treated with either PFOA or PFOS for 48 h. Results showed that both PFOA and PFOS were able to induce apoptosis in zebrafish liver cells.

p53, Bcl-2, Bax, caspase-3 and NF-κB p65 are key genes in the process of cell apoptosis. In the present study, we aimed to confirm that the mRNA and protein expression of these key genes in the pathway are altered and also aimed to further explore the effects of PFOA and PFOS in promoting apoptosis in the different dose groups.

The p53 protein plays a critical role in cell cycle arrest and apoptosis, and its loss or inactivation is common in human cancers [24,25,26,27]. Consequently, therapies to restore p53 function, particularly its ability to induce apoptosis, have been a research focus. p53 is a tumor suppressor gene, and contributes to DNA repair and cell apoptosis [28,29,30,31]. Moreover, p53 plays an important role in DNA damage and apoptosis induced by UV and chemical agents. Importantly, p53 has already been cloned in zebrafish and was confirmed to be expressed in the embryogenesis stage. Other studies identified the p53 pathway as the essential mediator of the elevated apoptosis in the developing zebrafish [32]. After stimulated with PFOA and PFOS, the gene expression of p53 increased. We attributed this to a transcriptional event because the mRNA level of p53 was induced upon treatment with PFOA-IC_50_, PFOA-IC_80_, PFOS-IC_50_ and PFOS-IC_80_ in ZFL cells (Figure 2). Consistent with an increase in mRNA, the protein level of p53 also significantly increased in PFOA-IC_50_ and PFOS-IC_80_ groups (Figure 5). These results indicated that PFOS and PFOA induced apoptosis in ZFL cells, presumably though the p53 pathway.

Some Bcl-2 family members are channel proteins and are located in the outer membrane of the mitochondria, endoplasmic reticulum and nuclear membrane, as well as the cytosol [33]. Depending on their function to inhibit or promote apoptosis, the Bcl gene family can be divided into apoptosis inhibiting genes (including Bcl-2 and Bcl-XL), and apoptosis promoting genes (including Bax, Bad and Bxl-XS) [34,35]. Prior to cell apoptosis, Bax is located within the cytoplasm. Upon receiving clues to induce cell death, Bax goes into the outer mitochondrial membrane and forms a specific channel to facilitate the release of cytochrome c. Conversely, Bcl-2’s functions are to prevent the release of cytochrome c in this process. Together Bax and Bcl-2 regulate and mediate the process by which mitochondria contributes to cell death, known as the intrinsic apoptosis pathway [36]. This pathway is required for preventing cancer and for normal embryonic development [36]. In our study, Bcl2 was found to be highly expressed in ZFL cells, whereas the expression of Bax was relative low. ZFL cells treated with PFOA and PFOS expressed higher levels of Bcl-2 (Figure 2 and Figure 5). Anti-apoptotic response were made, and activated the expression of Bcl2. However, large amounts of apoptosis cell were eventually proved and detected by flow cytometry, indicating that Bcl2 mRNA is not effectively translated into apoptotic inhibition proteins.

Members of the caspase family of aspartic acid-directed cysteine proteases lead to the loss of cellular structure and function and eventually result in apoptotic cell death [37,38]. In mammalian cells, the caspase family is comprised of at least 14 enzymes, which can be divided into two categories: initiator caspases and executioner caspases, depending on where they function in the apoptotic cascade [39]. Caspase-3, the essential effector caspase, plays a pivotal role during caspase-dependent apoptosis. The main function of caspase is to hydrolyze proteins and connect the caspase cascade in the upstream and downstream apoptotic process. Amino acids of caspase-3 in zebrafish have great homology with vertebrates. In our study, the mRNA level of caspase 3 was induced with the treatment of PFOA and PFOS (Figure 2), and the protein level of pro-caspase 3 increased in the PFOA-IC_50_ group and the PFOS-IC_80_ group (Figure 5). These results revealed that PFOS and PFOA induced apoptosis in ZFL cells with involvement of caspases [22].

To sum up, this study confirmed the crucial genes in the pathway of apoptosis and further illustrated the effects of PFOA and PFOS on the process of apoptosis. It provides a theoretical basis for future study of cell apoptosis.

## 5. Conclusions

According to the results in this study, we noted that PFOA-IC_80_ and PFOS-IC_50_ both promoted apoptosis. The decreased percentage of cells in G1/G0 stage and the increased percentage of cells in G2/M stage clearly showed that cell cycle and cell proliferation were blocked in both the PFOA-IC_80_ and PFOS-IC_80_ groups. This study confirmed the crucial genes in the pathway of apoptosis and further illustrated the effect of PFOA and PFOS in the process of apoptosis.
